# Supplementary figures and images for: The RNA-binding protein Msi2 regulates autophagy during myogenic differentiation
Source: Life Sci Alliance. 2024 Feb 19;7(5):e202302016. doi: 10.26508/lsa.202302016 (PMC10876439; doi:10.26508/lsa.202302016)

**A**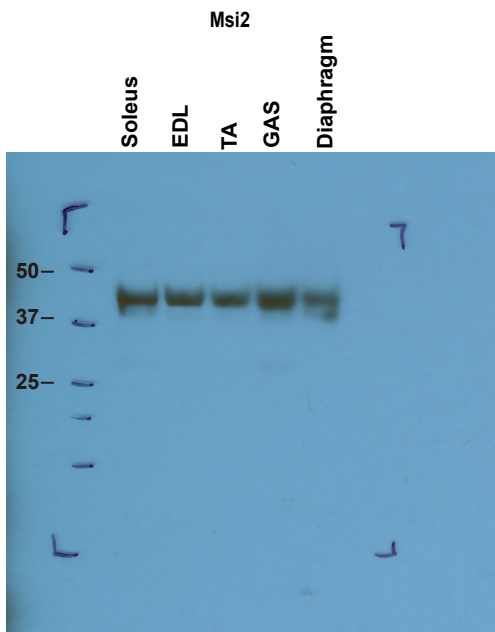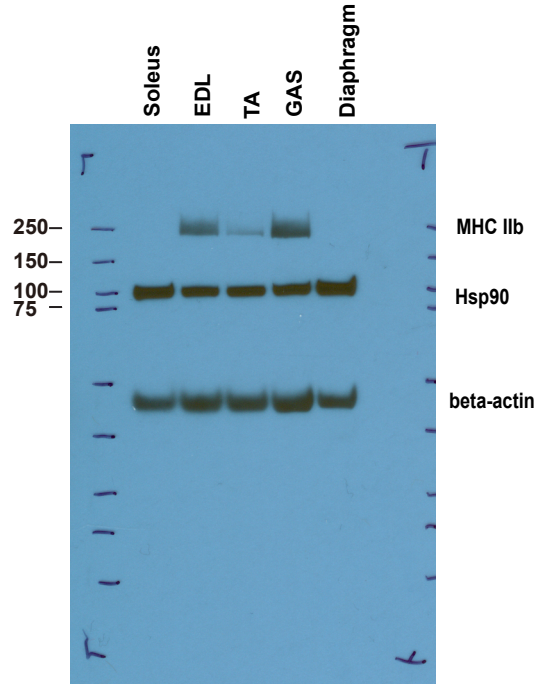**D**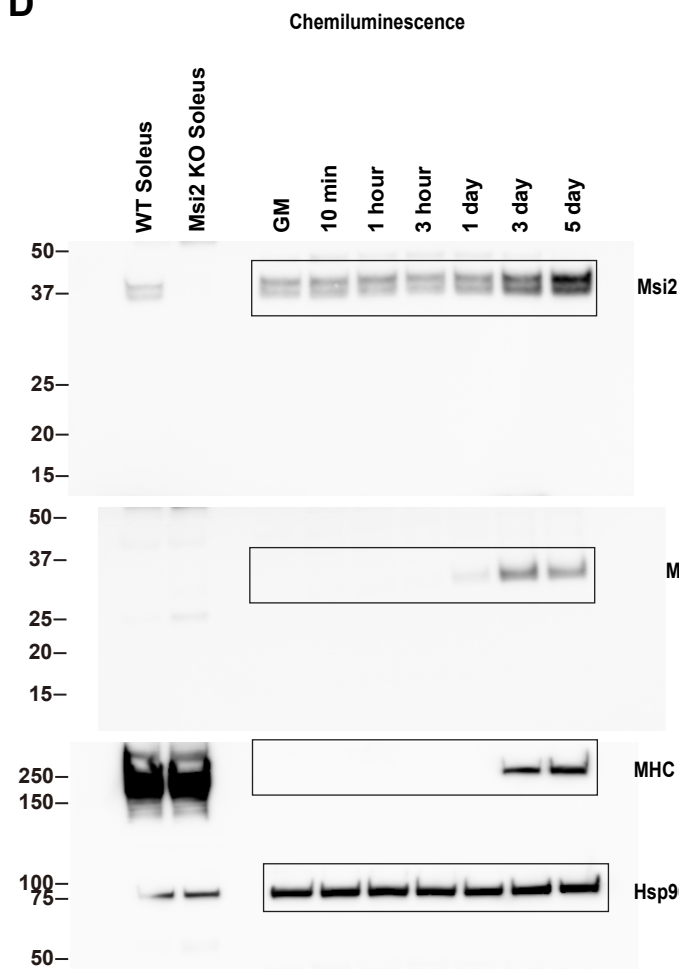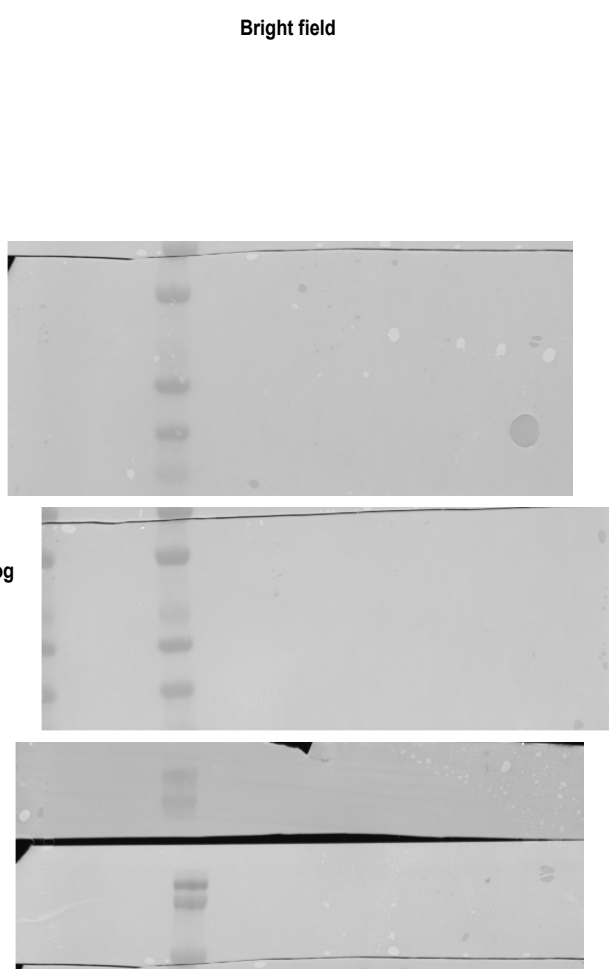

Supplement: Supplementary file 1 [file LSA-2023-02016_SdataF1.pdf]

**A**

Chemiluminescence

Bright field

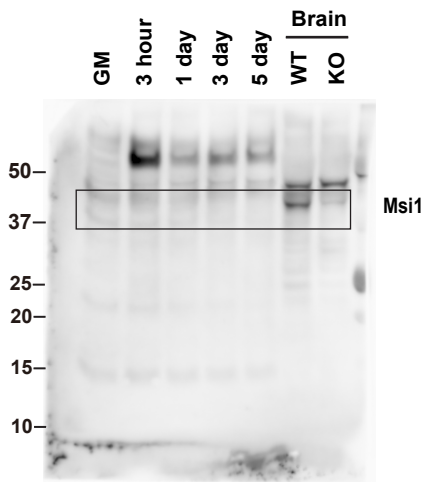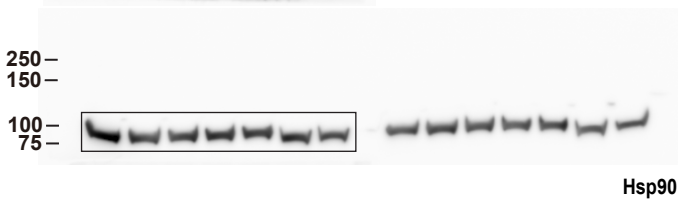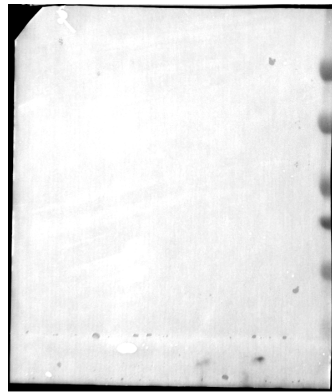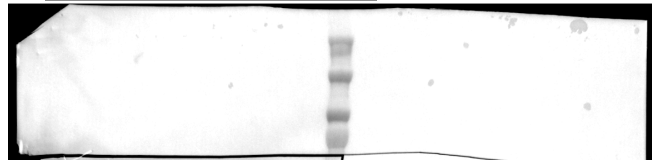

Supplement: Supplementary file 2 [file LSA-2023-02016_SdataFS1.pdf]

**A****Chemiluminescence****Bright field**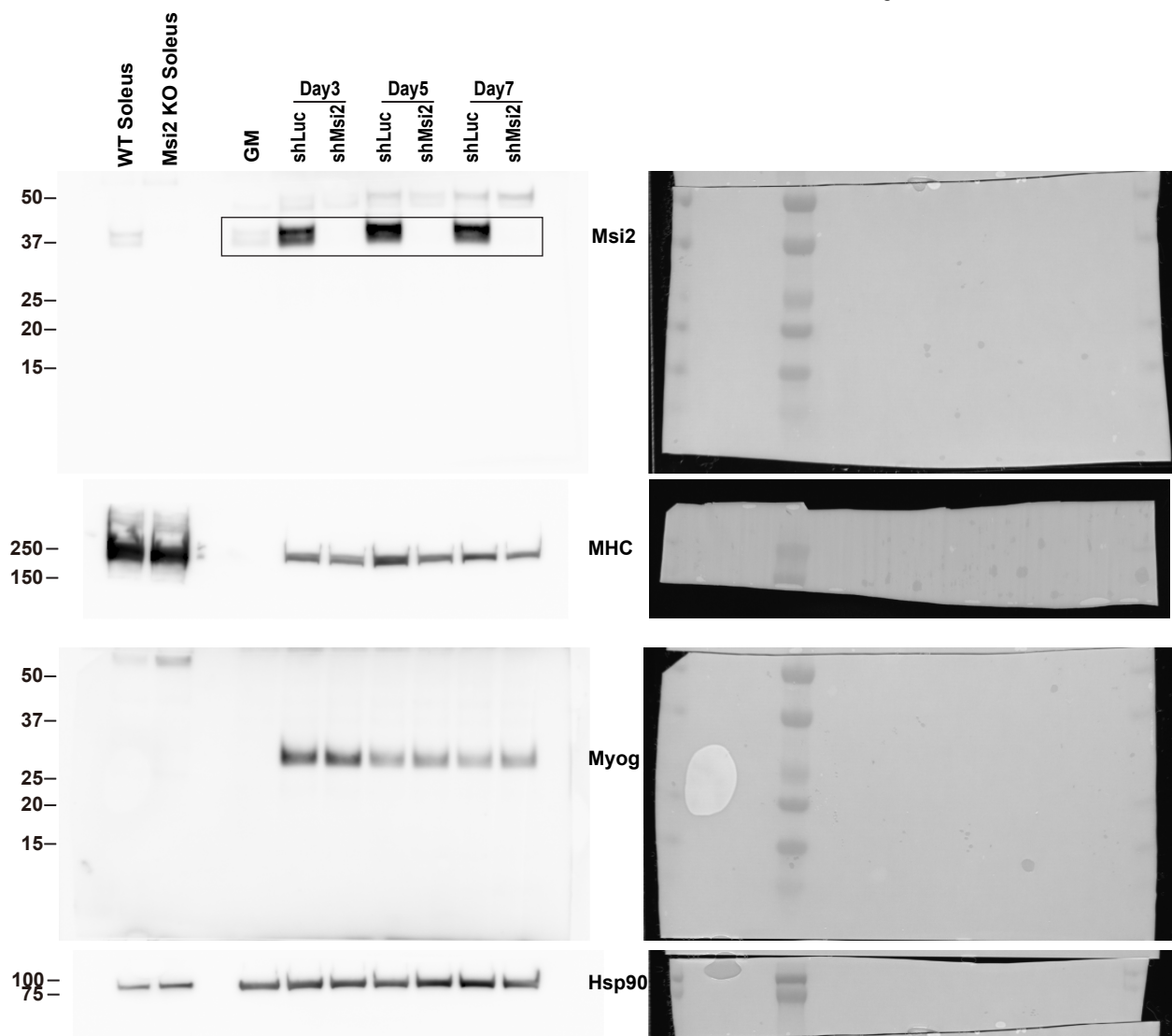

Supplement: Supplementary file 3 [file LSA-2023-02016_SdataF2.pdf]

**C**

Chemiluminescence

Bright field

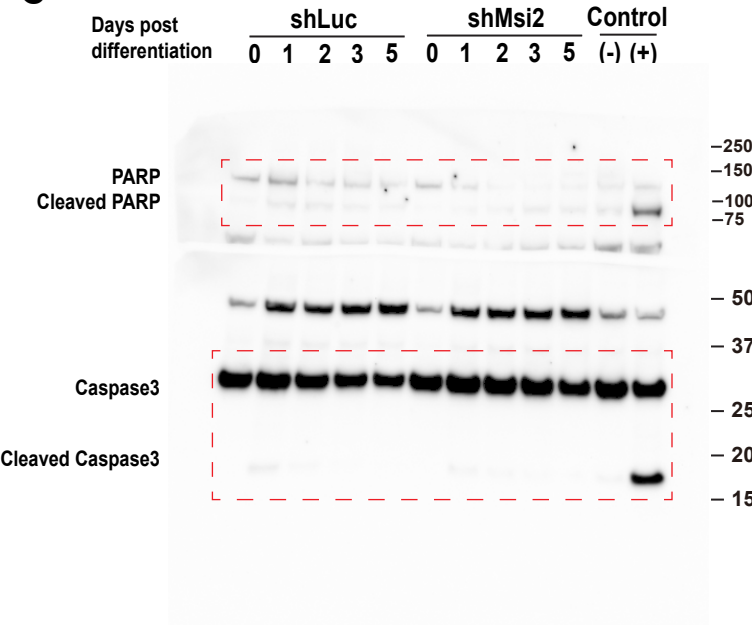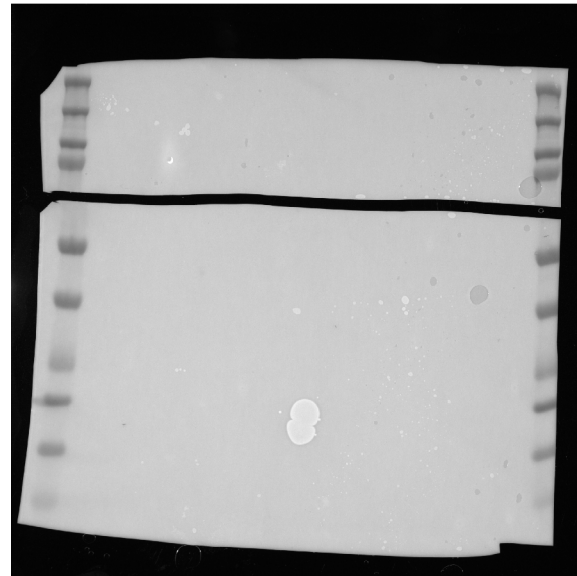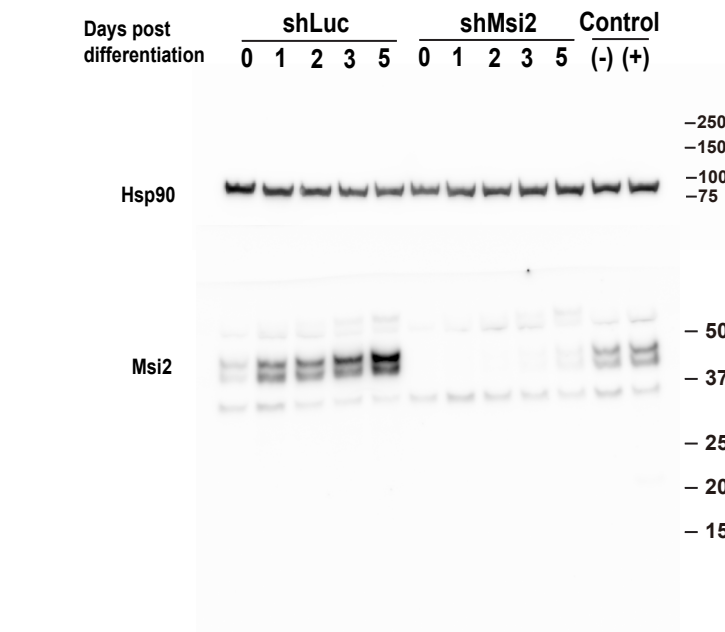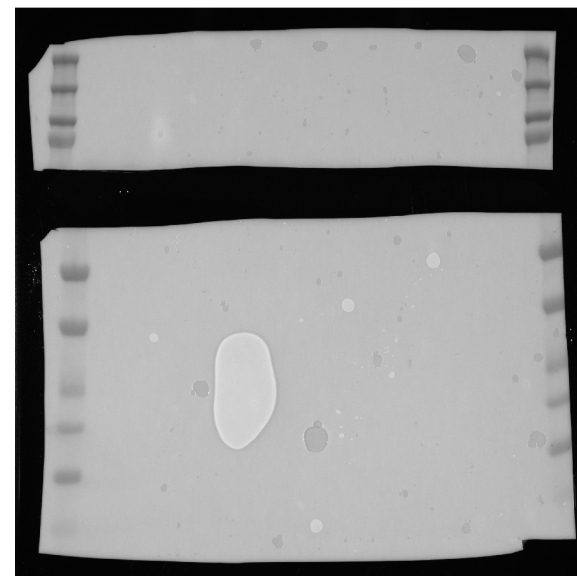

Supplement: Supplementary file 4 [file LSA-2023-02016_SdataFS2.pdf]

F

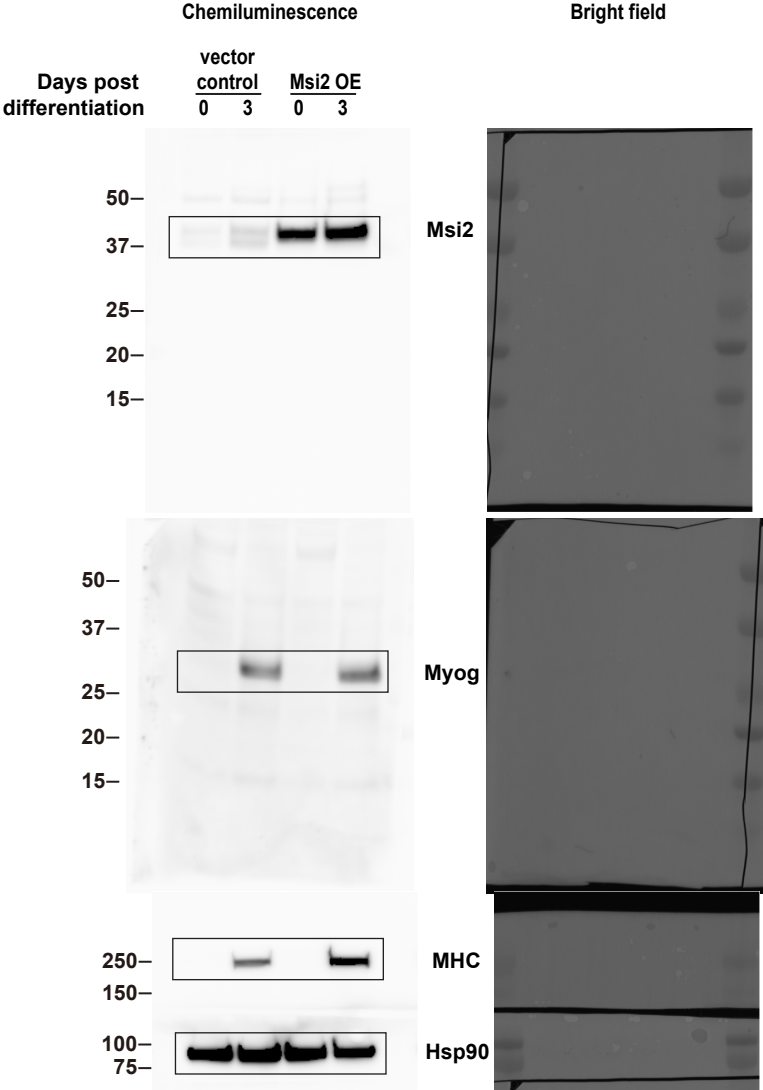

Supplement: Supplementary file 5 [file LSA-2023-02016_SdataF3.pdf]

**B**

Chemiluminescence

Bright field

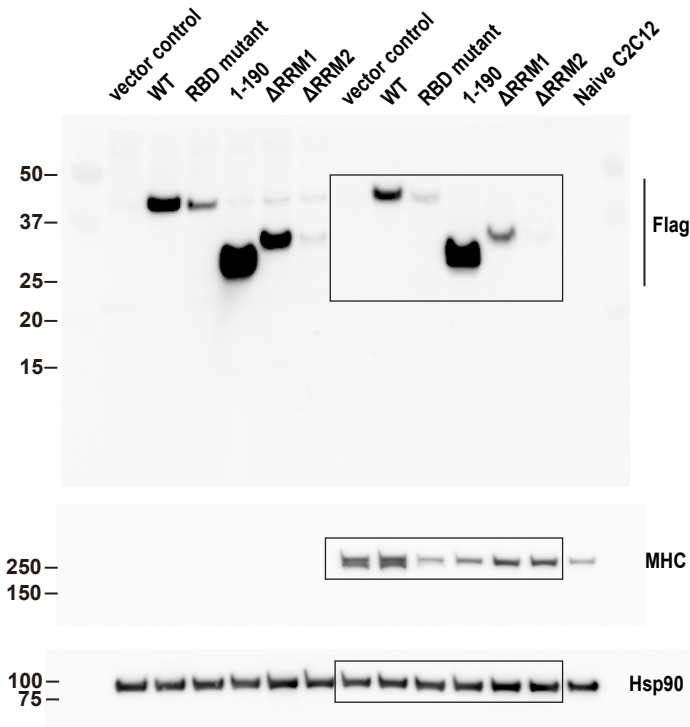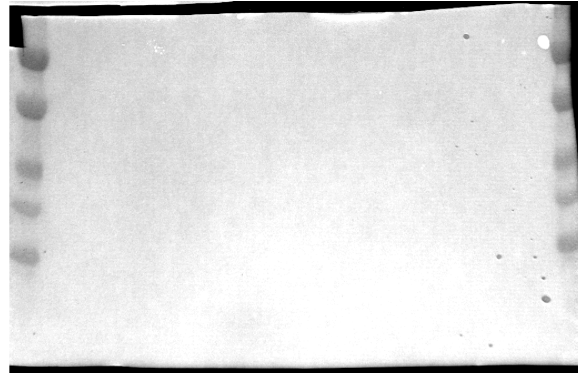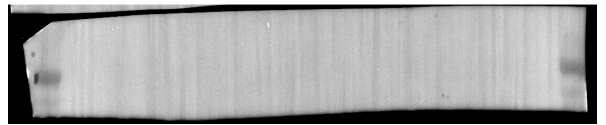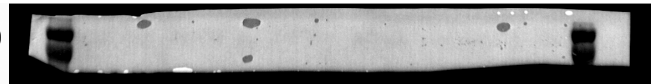

Supplement: Supplementary file 6 [file LSA-2023-02016_SdataF4.pdf]

**A**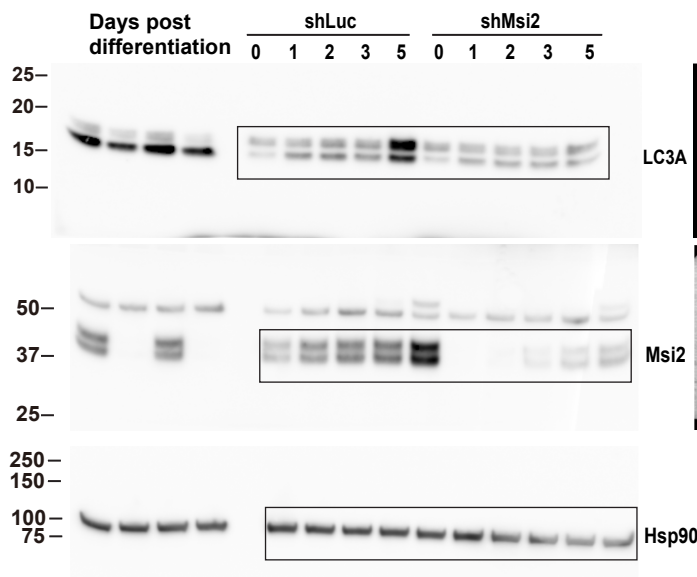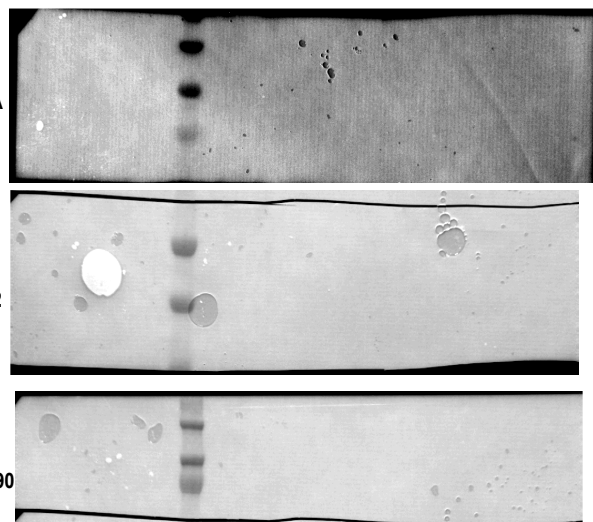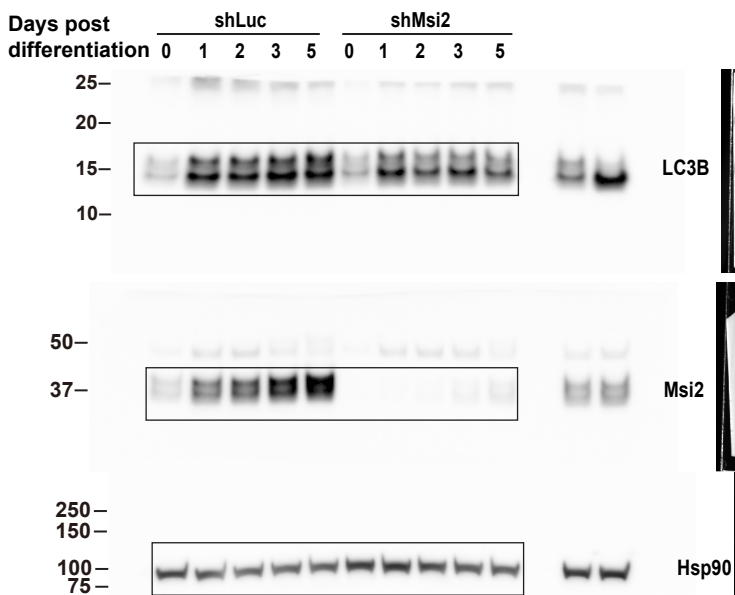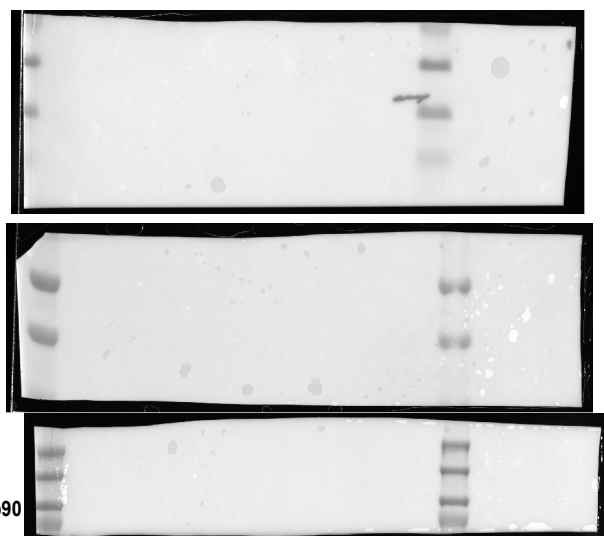

Supplement: Supplementary file 7 [file LSA-2023-02016_SdataF5.pdf]

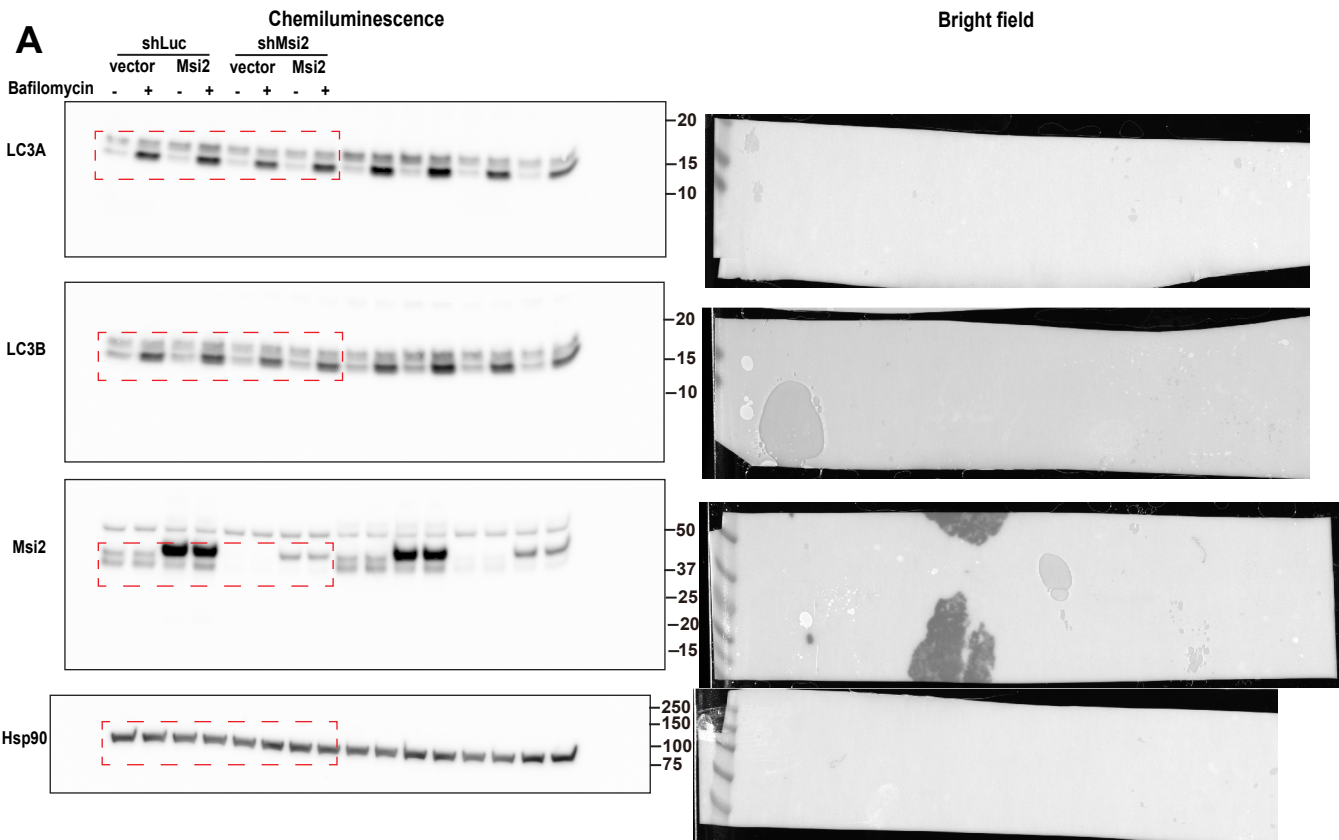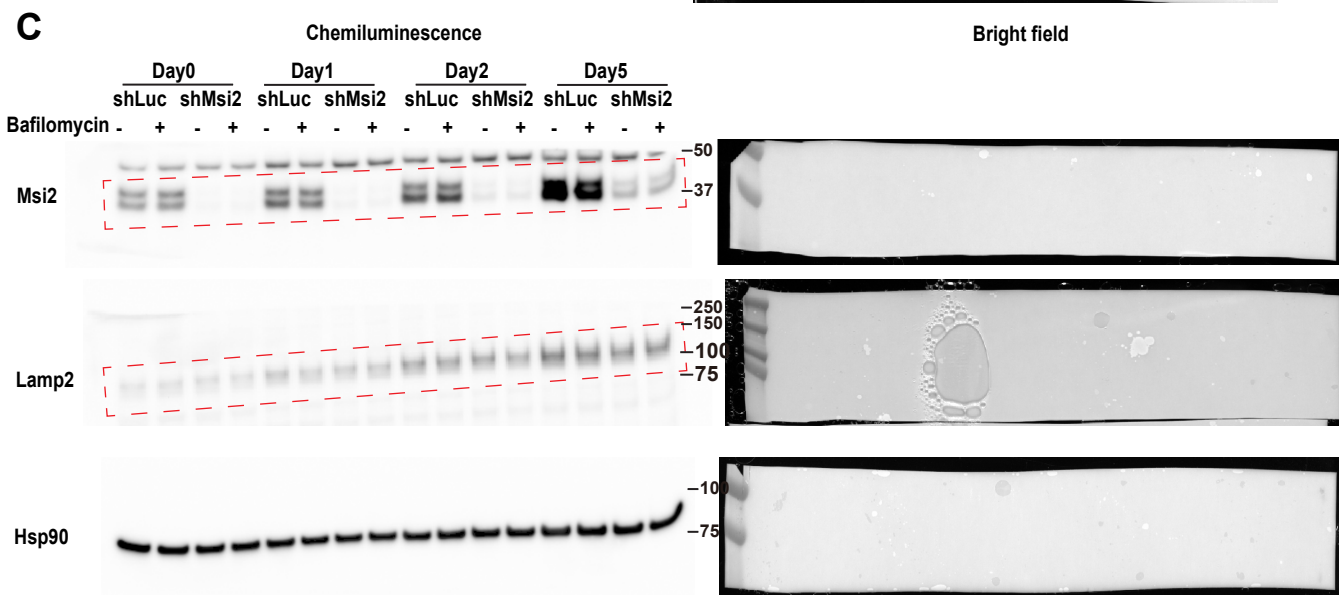

Supplement: Supplementary file 8 [file LSA-2023-02016_SdataFS4.pdf]
