# Supplementary material for: The RNA-binding protein Msi2 regulates autophagy during myogenic differentiation
Source: Life Sci Alliance. 2024 Feb 19;7(5):e202302016. doi: 10.26508/lsa.202302016 (PMC10876439; doi:10.26508/lsa.202302016)
Supplement: Supplementary file 9 [file LSA-2023-02016_SdataF6.pdf]

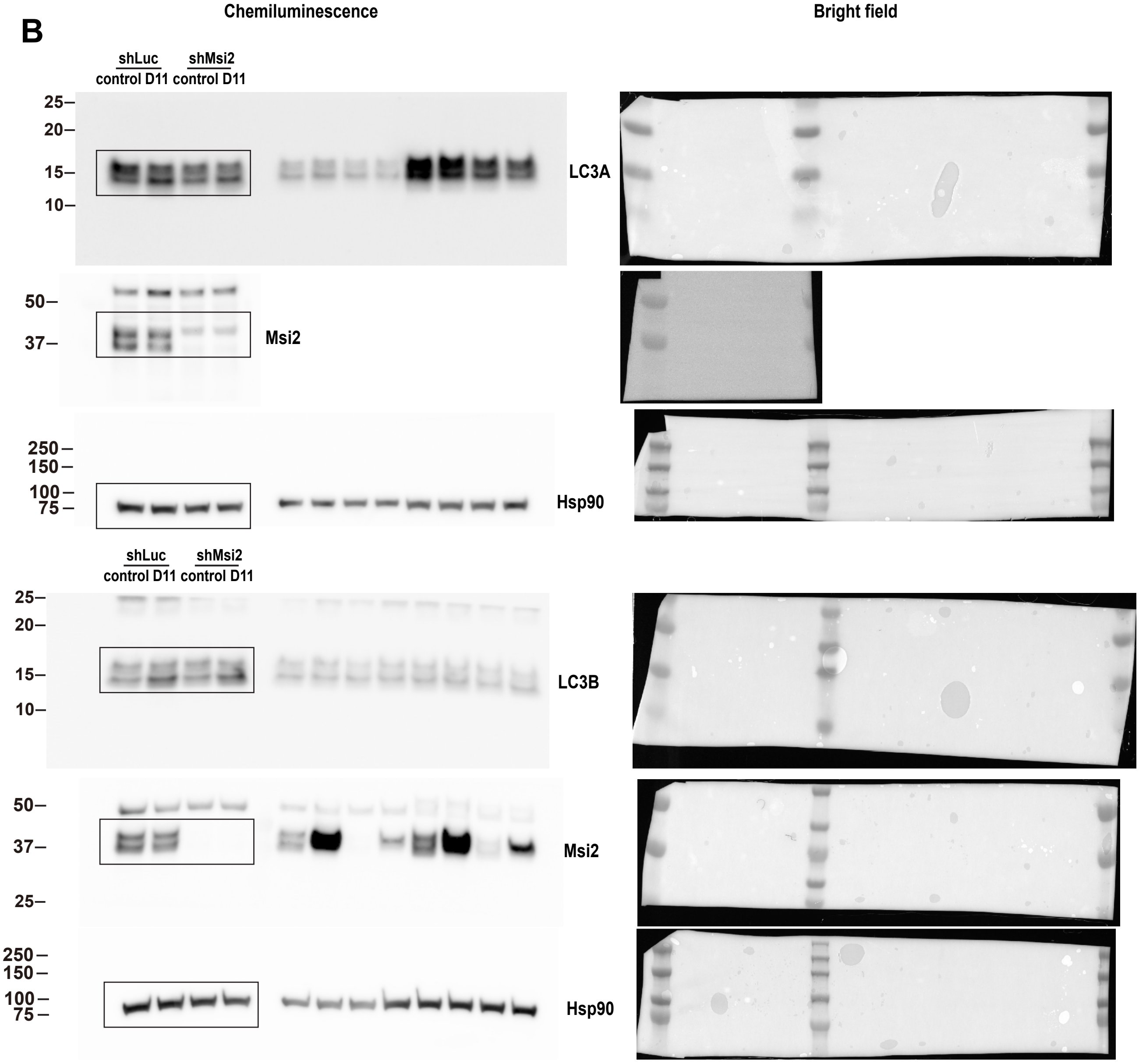
B
Chemiluminescence
Bright field
shLuc
shMsi2
control D11 control D11
25
20
15
10
LC3A
50
37
Msi2
250
150
100
75
Hsp90
shLuc
shMsi2
control D11 control D11
25
20
15
10
LC3B
50
37
25
Msi2
250
150
100
75
Hsp90
