# Supplementary material for: The RNA-binding protein Msi2 regulates autophagy during myogenic differentiation
Source: Life Sci Alliance. 2024 Feb 19;7(5):e202302016. doi: 10.26508/lsa.202302016 (PMC10876439; doi:10.26508/lsa.202302016)
Supplement: Supplementary file 10 [file LSA-2023-02016_SdataFS5.pdf]

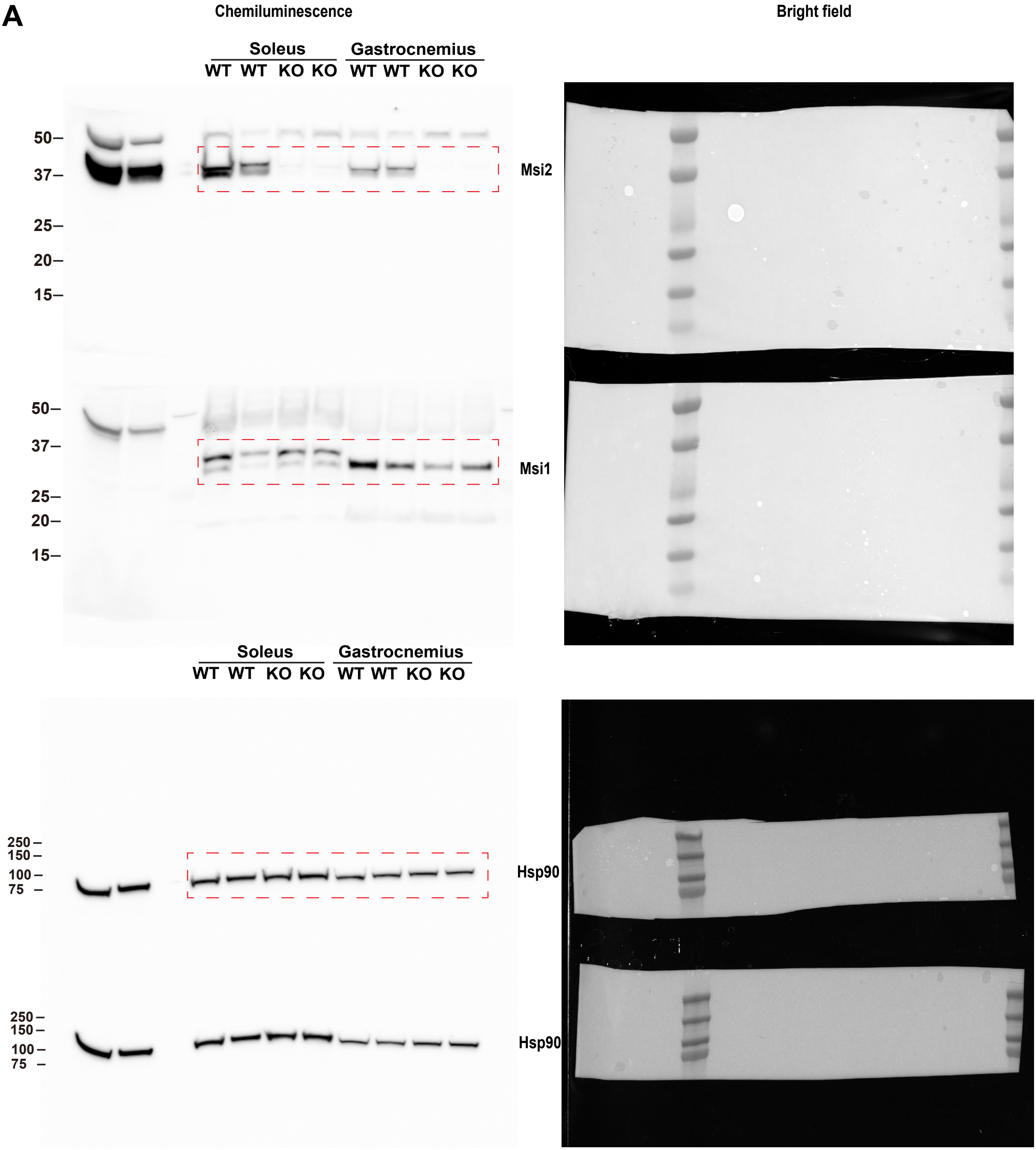
A
Chemiluminescence
Bright field
Soleus
Gastrocnemius
WT WT KO KO WT WT KO KO
50–
37–
25–
20–
15–
Msi2
50–
37–
25–
20–
15–
Msi1
Soleus
Gastrocnemius
WT WT KO KO WT WT KO KO
250 –
150 –
100 –
75 –
Hsp90
250 –
150 –
100 –
75 –
Hsp90
